# Language play facilitates language learning: Optimizing the input for gender-like category induction

**DOI:** 10.1186/s41235-016-0038-z

**Published:** 2017-02-20

**Authors:** Johanna Bebout, Eva Belke

**Affiliations:** Sprachwissenschaftliches Institut, Ruhr-Universität Bochum, D-44780 Bochum, Germany

**Keywords:** Language learning, Language play, Grammatical gender, Language acquisition

## Abstract

Gender induction has been claimed to be virtually impossible unless nouns provide reliable semantic or phonological gender-relevant cues. However, learners might exploit syntactic cues, such as definite articles, to infer the gender of gender-unmarked nouns. In children’s poems and songs, such syntactic cues are presented in a highly structured fashion. We assessed gender-like category induction in an artificial language that provided exclusively syntactic cues for its gender-like subclasses. We trained participants with structured or unstructured input presented in a prose, a rhyming, a melodic, or a rhyming and melodic fashion. Input structuring significantly facilitated gender-like category induction. Participants trained in the Rhyme-and-Melody mode significantly outperformed participants trained in the Prose mode, especially when the input was structured. The Rhyme-only and Melody-only modes yielded intermediate results. Thus, a highly structured rhyming and melodic input substantially facilitates gender-like category induction, making a case for the use of children’s songs in language teaching.

## Significance

For learners of German, especially those learning German as a second language, the correct use of determiners constitutes a major challenge. They appear to struggle most with the correct assignment of nouns to gender subclasses, which is rather opaque in German. Mastering the gender assignment of nouns is key to sentence comprehension, so failing to acquire it can have substantial knock-on effects on academic success. Given that about a quarter of the children in Germany acquire German as a second language, it is crucial that they are optimally supported during their acquisition process at an early age.

We investigated how the acquisition of opaque gender-like noun subclasses seen in German and several other languages, such as Russian, can be facilitated by means of input optimization. As a starting point, we considered language play, as seen in children’s poems, songs, or nursery rhymes. They are highly structured, structurally repetitive, and often combine a highly structured input with a rhyming or a melodic presentation, or both. Arguably, these characteristics render language plays an excellent starting point for language learning and teaching but, as yet, this claim has not been verified experimentally. In an artificial language learning study, we demonstrate that input structuring, rhyme, and melody are effective means to enhance the acquisition of gender-like subclasses.

Our findings make a clear case for using children’s songs and poems in language training and teaching. Most likely, they apply across languages to other grammatical categories marked as opaquely as German gender.

## Introduction

One of the challenges of language learning is to acquire knowledge about how verbs and nouns in an unfamiliar language are inflected. It requires the learner to find out about the relevant markers for inflection, also called morphemes, and about which words are associated with which sets of inflectional morphemes. Regular verbs in English, for instance, are associated with a specific set of morphemes, often referred to as a paradigm, that are required to indicate information on tense, number, and person, such as -s, -ed, -∅ in *I paint-∅*, *he/she paint-s*, *she paint-ed*.

Gender-marking languages, such as French, Italian, German, or Russian, typically feature different paradigms of morphemes for each grammatical gender. For example, Table 1 presents the three paradigms of definite articles in German associated with each gender subclass (masculine, feminine, or neuter singular) to mark the four cases of singular nouns (nominative, genitive, dative, and accusative). The number of gender subclasses and the rules for assigning nouns to these subclasses differ considerably between languages.

**Table 1:** The definite articles in the German gender-case paradigm (singular only) for nominative, genitive, dative and accusative cases

Case	Gender category		
	Masculine	Feminine	Neuter
Nominative	der	die	das
Genitive	des	der	des
Dative	dem	der	dem
Accusative	den	die	das

When acquiring a gender-marking language, a learner can make use of a variety of cues towards a noun’s gender. *Syntactic cues* are available in the morphosyntactic environment of a noun, including gender-marked morphemes, such as definite articles or pronouns that co-occur with a noun. In Italian, for example, the definite articles “il” and “la” are strong cues that the nouns they accompany are masculine and feminine, respectively (Danesi, 2012). *Noun cues*, by contrast, are cues based on a noun’s meaning or form, i.e., its phonology or morphology. In Italian, the phonological forms of many nouns provide strong cues towards their gender; for instance, nouns ending in -a are feminine and nouns ending in -o are masculine in most of the cases (Danesi, 2012). In German, derivational morphemes, such as -ung, -heit or -er (see (1) below), which turn a verb or an adjective into a noun, constitute one of the few unambiguous noun cues in the language; in fact they determine the gender of the derived form, which is feminine for -ung and -heit, and masculine for -er:

Gender systems of languages such as Italian, which provide many unambiguous noun cues towards the gender of their nouns, are often described as being relatively easy to acquire compared to languages such as German, whose gender system provides few unambiguous noun cues requiring the learners to make increased use of syntactic cues (Brooks, Braine, Catalano, Brody, & Sudhalter, 1993; MacWhinney, Leinbach, Taraban, & McDonald, 1989; Wegener, 1995).

In the present study, we aimed to find out whether optimizing the presentation of the input during training can facilitate the acquisition of gender assignment in a gender-marking language which provides exclusively syntactic and no noun cues. As a starting point for our research we considered language plays, i.e., aesthetic texts, such as poems, songs, or nursery rhymes, that are produced in playful situations (E. Belke & G. Belke, 2006). Language plays are characterized by being highly structured and structurally repetitive, often featuring the same sequence of utterances over and over again with varying agents or objects. This distinguishes them from everyday interaction, which is structurally rather variable. Moreover, language plays often combine a highly structured input with a rhyming or a melodic presentation or both. It has been argued that these characteristics render language plays an excellent starting point for language learning and teaching (see G. Belke, 2007, 2012; E. Belke & G. Belke, 2006; Cook, 1997, 2000; Haueis, 1985). However, experimental evidence to support this claim is scarce. We studied the acquisition of artificial gender-like subclasses in two groups of participants, with one being trained with highly structured input such as that seen in language plays and the other receiving the same training sentences in an unstructured training regime, mimicking verbal interactions in everyday life. For both groups, we tested the effect of rhyme and melody—key features of nursery rhymes and children’s songs—on participants’ performance.

In the remainder of this introduction, we will first review evidence suggesting that the acquisition of gender assignment systems with very few or no phonological but only syntactic cues is extremely hard but possible, provided that the input is structured in a learner-friendly fashion. In doing so, we introduce the artificial language learning paradigm used for studying the acquisition of gender-like subclasses. We shall then turn to German, a gender-marking language with three gender subclasses whose gender assignment system has been described by some as being largely arbitrary for its small number of valid noun cues (Maratsos, 1983, but see Köpcke, 1982). In the final section, we make the case for why language play in general, and children’s songs in particular, are a means of presenting syntactic cues in an optimal way.

### Cues in gender-like category induction

In a seminal study, Brooks et al. (1993) hypothesized that the acquisition of artificial gender-case-like subclasses might be impossible unless the nouns are phonologically or semantically marked for gender. They trained undergraduate students with an artificial language under two conditions. In the experimental condition, the nouns were assigned to two grammatical subclasses (Subclass 1 and Subclass 2) in such a way that the phonological form of most of the nouns was informative regarding their assignment to gender subclasses. In the control condition, by contrast, the nouns were assigned randomly to the two subclasses.

Every noun referred to an object, such as “hoik” for house, and was combined with other elements of the artificial language to form sentences. These sentences described how an actor, “Frippy”, moved relative to the object depicted by the noun. All sentences included the agent, “Frippy”, an object noun, and a locative suffix (either “eef”, “rog”, “ast”, “foo”, “ilg”, or “tev”). The suffixes indicated one of three different prepositions (to, at, or from) relating to the direction or location of “Frippy” relative to the object. For instance, the sentence for “Frippy” walking towards a house (“hoik”) would be “Frippy hoik-eef”. The suffix paradigm for the prepositions to, at, and from differed depending on the grammatical subclass of the object noun. For example, the preposition *to* translated to “eef” for nouns of Subclass 1, but to “foo” for nouns of Subclass 2. Hence, in both the experimental and the control condition the suffix paradigm presented reliable information about the subclass of the noun with which each suffix was associated. The conditions differed, however, with respect to the availability of additional phonological cues at the noun.

One experimental and one control group were exposed to the language in four or five training sessions involving comprehension and production tasks. In the test session, the experimental group yielded significantly better results in comparison to the control group with regard to both tests, suggesting that a combination of noun cues and syntactic cues is sufficient to enable participants to acquire grammatical subclasses in an artificial language. Critically, the performance attained by the control group did not exceed chance, indicating that syntactic cues alone are insufficient for acquiring information on grammatical subclasses. In Experiment 2 of their study, the authors replicated their findings with 9- and 10-year-old children. Taken together, these findings provide evidence for noun-cue models of gender acquisition, according to which phonological or semantic noun cues are indispensable for acquiring a noun’s gender. Subsequent studies have confirmed the central role of noun cues (e.g., Frigo & McDonald, 1998; Kempe & Brooks, 2008; Monaghan, Chater, & Christiansen, 2005; Monaghan, Christiansen, & Chater, 2007; Taraban, 2004; Taraban & Kempe, 1999).

However, Taraban (2004; Experiment 3) showed that the acquisition of phonologically and semantically unmarked gender subclasses is possible if the learners’ attention is drawn towards the relevant syntactic context. In his study, he employed the same language as Brooks et al. (1993), making sure that there were no noun cues, i.e., all nouns were phonologically unmarked for their gender subclass. In one condition, the blocked condition, Taraban (2004) presented the input in such a way that all phrases pertaining to one noun were shown in immediate succession. For example, the three to, at, and from phrases for the noun “billit” (ball) were presented as a group, allowing participants to process the whole paradigm of morphemes pertaining to this noun. This way, their attention was guided towards the relevant markers in the syntactic context. In a second, random condition, Taraban exposed participants to all phrases in random order, as had Brooks et al. (1993).

The results showed that the performance of the blocked group was significantly better than that of the random group. Interestingly, the advantage for the blocked group emerged only when the lexicon was about as large as the one Brooks et al. had used (including 11 words per subclass). There was no such advantage for a small lexicon with only four words per subclass. This suggests that, provided the lexicon to be acquired is of a reasonable size, a blocked presentation is sufficient for drawing the attention towards the underlying lexical subclasses of the nouns.

### Gender assignment in German

German presents an interesting case in the light of the findings reported by Brooks et al. (1993), as it provides only very few valid noun cues. There is only one set of highly valid noun cues associated with the derivational morphemes reviewed in (1). Note, however, that these cues typically occur in rather abstract and long words that are unlikely to be part of the vocabulary of young children.^1^ All other semantic, morphological, or phonological gender-assignment regularities have many exceptions. This is partly due to the fact that the regularities override each other: morphological regularities override semantic regularities, such as the natural gender principle,^2^ and semantic regularities override phonological regularities (see Wegener, 1995). For instance, “Mädchen” (girl) (see (2) below) should be assigned feminine gender by the natural gender principle; however, this semantic regularity is overruled by the stronger morphological rule that the diminutive suffix “-chen” is associated with neuter gender.^1^ Similarly, by its phonological form, “Junge” in (2) should be assigned female gender because it is disyllabic and ends in /ə/; however, due to the natural gender principle,^2^ it is assigned masculine gender.

Due to their position at the bottom of the hierarchy, phonological regularities are particularly volatile. Köpcke (1982) (see also Köpcke & Zubin, 1984) identified 24 phonological gender assignment regularities for 1466 monosyllabic nouns in German. Some of the regularities are quite valid but apply to only a very small number of words. For instance, the regularity that nouns beginning with /kn/, as in *Knopf* (button.M) or *Knall* (bang.M), are masculine applies to only 15 out of 1466 monosyllabic words. Furthermore, about half of the regularities identified by Köpcke assign either masculine or neuter gender, hence only ruling out the feminine gender. *Not* assigning the feminine gender to a monosyllabic word is the default anyway, as most of the monosyllabic nouns Köpcke analyzed were either masculine (940 words) or neuter (321 words), exceeding the number of feminine words (205 words) by 4.6 and 1.6 respectively (see Köpcke, 1982).

Revising and extending Köpcke’s work, Wegener (1995) put forward a smaller set of gender assignment rules that apply to not only monosyllabic but also multisyllabic words (see Table 11 in Appendix 1). One of these rules is that monosyllabic nouns have a masculine gender by default. This rule covers two-thirds of the nouns Köpcke had analyzed; however, it also leaves one-third of the nouns unaccounted for. Given the difficulties in establishing valid gender assignment rules for monomorphemic words in German, some authors have argued that, from the learners’ perspective, these words are largely arbitrarily assigned to gender subclasses (Maratsos, 1983).^3^

Indeed, compared to noun cues, the gender of German monomorphemic nouns appears to be more reliably indicated by syntactic cues in the morphosyntactic environment of the noun, such as the definite and indefinite determiners and other gender-marking morphemes (e.g., pronouns) (MacWhinney et al., 1989; Schwichtenberg & Schiller, 2004). This is consistent with syntactic-context models, according to which the morphosyntactic context plays a pivotal role when acquiring gender (Maratsos & Chalkley, 1980; Taraban, 2004). According to these models, gender categories are learnable through the combination of the noun with small sets of associated marker morphemes (e.g., the articles in the German masculine singular form (*der*, *des*, *dem*, *den*; see Table 1) plus the associated masculine singular pronouns *er* (‘he’), *sein* (‘his’), *ihm* (‘him’), *ihn* (‘him’)). Note, however, that in German individual markers, such as “il” or “la” in the Italian case, may be misleading gender assignment cues: some markers feature across gender subclasses, as shown in Table 1 for the case of German definite articles. While each gender subclass is associated with a distinct case marking paradigm of definite articles, the full set of 12 grammatical functions (three genders by four cases) is served by only six different forms; “der”, for instance, occurs in two different genders and three different cases.

Despite these difficulties, most children with German as their native language master the gender system by the age of three (Szagun, Stumper, Sondag, & Franik 2007). Critically, however, there is a large proportion of children in Germany who acquire German as a second language. These children experience apparent difficulties during the acquisition of the German gender-case system (e.g., Jeuk, 2007; Rösch, 2003), producing incorrectly gender- and case-marked determiners, adjectives, and pronouns. Jeuk (2007) observed that for second language learners aged six and older, the correct use of German determiners constitutes a major challenge with the most pressing problem being the correct assignment of nouns to gender subclasses. The reasons for these problems are probably manifold. One is that children growing up with German as their second language are not exposed to as frequent and as rich a German input in their early childhood as their monolingual peers. A second one is that they may have a harder time making use of the cues that are available in the input, simply because their mastery of German is less advanced than that of their monolingual peers.

Mastering the gender-case system is key to sentence comprehension. For instance, in order to establish the referent of a pronoun, gender is a most relevant cue. Take the two sentences in (3) (taken from Köpcke & Zubin, 1984, p. 43): In (3a) the pronoun “sie” refers unambiguously to the bowl, in (3b) the pronoun “er” refers unambiguously to the jug:

Note that in (3b) the referent of “er” (it.M) is not the noun immediately preceding the pronoun but a noun further away. Such long-distance pronominal references can only be decoded when the gender-number agreement of the pronoun and its referent is evaluated correctly. They are particularly prevalent in complex written texts, which is why failing to fully acquire the gender-case (and number) system of German can have knock-on effects on text comprehension and, ultimately, on academic success. Given that about a quarter of the children in Germany acquire German as a second language (Authoring Group Educational Reporting, 2016) it is crucial that these children are optimally supported during their acquisition process at an early age.

### Language play as input optimization?

So far, we have established that assigning the correct gender to nouns presents a major problem for many children in Germany, especially for those growing up with German as a second language, and that this may impact negatively on their ability to understand complex written texts. Given that syntactic cues represent the most reliable hint as to the gender of a German noun, we asked whether there is a way to optimize the presentation of the relevant syntactic cues for language teaching and intervention purposes. We looked at language play as a starting point, for it has many features beneficial to language learning, including input structuring, repetition, and repeatablility, upon which we want to focus here.

First, language plays, such as songs, tongue twisters, or nursery rhymes, typically include many repetitions and can be repeated many times at no pragmatic cost. Cook (1997, 2000) points out that young children in particular enjoy repeated patterns and like to listen to the same stories again and again without getting bored. Additionally, children like to produce language plays by heart (Cook, 1997). By repeating a text over and over again, the time for language processing as well as the text’s predictability increases (Cook, 2000).

Many nursery rhymes, children’s songs, and also children’s stories repeat grammatical structures while only making few lexical substitutions, facilitating the detection and isolation of individual linguistic units (G. Belke, 2012). Cook (2000) argues that one function of such parallelisms across repetitions might be to draw “attention to individual words, while also illustrating their occurrence in common collocations and colligations” (p. 30). In Appendix 2, we present an adapted version of a well-known German children’s song by Fredrik Vahle (1988) as an example. In this song, a variety of animals approach a cat and ask it to dance, but the cat finds all kinds of excuses for why it would rather dance alone. Critically, in each verse of the adapted version, the animal approaching the cat is mentioned in three different grammatical cases—nominative, accusative, and dative—establishing the three relevant cases needed for identifying a paradigm of articles unique to the grammatical gender of the animal (e.g., Igel (hedgehog.M): der–dem–den; Ente (duck.F): die–der–die; Schwein (pig.N): das–dem–das; cf. Table 1 and Appendix 2). Hence, in this case, the verse structure introduces a structured presentation of the gender-relevant syntactic context of the animals while the lexical content is kept rather simple and repetitive (cf. Frieg, Hilbert, E. Belke, & G. Belke, 2012; for more text examples and songs, see G. Belke, 2007; Kauffeldt et al., 2014).

It is noteworthy that the presentation of the nouns in this version of the children’s song is akin to the blocked input presentation employed by Taraban (2004). Critically, in the children’s song, the relevant linguistic input is presented in a rhyming, melodic, and rhythmic presentation. It would seem intuitive that melody, rhythm, and rhyme enhance the intensity of engaging with a text and render language learning even more effective overall. However, to date, there is only very little research to prove or disprove this intuition, as we will review in the following sections.

#### Effects of rhyme and melody on utterance likeability

Children’s songs and rhymes are easy to recognize for their rhythmicity and lyrical simplicity, their repetitiveness and, in the case of songs, the simplicity of their tunes. Children seem to like these features and enjoy singing and playing with language. It is conceivable that increased likeability leads participants to process the linguistic input more attentively and memorize it better. In a study with adult speakers, Obermeyer et al. (2013) showed that rhyming and metered speech is perceived as more likeable than prose speech. Using five-point Likert scales, they assessed real-word and pseudoword variants of rhyming and metered poems in comparison to non-rhyming, non-metered, and non-rhyming and non-metered variants of the poems in terms of a) the participants’ liking ratings (very bad to very good), b) the self-rated intensity of their emotional response (weak to strong), c) the emotion they perceived from the spoken text, and d) their felt emotion (very negative to very positive). Meter and rhyme increased the liking ratings and the intensity ratings. Participants rated their felt emotion higher for rhyming than for non-rhyming texts, especially when listening to the pseudoword variants of the poem. These findings suggest that, like melody, rhyme can boost a positive emotional response and the likeability of a spoken text. Metered text may be perceived as more likeable than non-metered text because its pitch contours present a protoform of a melodic presentation (Heffner & Slevc, 2015; Lehrdahl, 2001).

#### Effects of rhyme and melody on language processing

In terms of processing metered, rhyming, or even sung speech, there is evidence that in metered speech syllables spoken on the beat are perceived more effectively than syllables produced off the beat. Cason and Schön (2012) showed that French participants engaged in a phoneme monitoring task detected phonemes faster when they were produced in a syllable on the beat rather than off the beat, regardless of the type of meter (binary: weak–strong, ternary: weak–weak–strong). They also found that participants primed with the binary or ternary meter displayed a P300 when the target word did not match that meter, indicating that participants processed the meter of prime and target. Cason, Astésano, and Schön (2015) report parallel results, with participants performing better in a phoneme judgment task when the target utterance matched rhythmically with a prime than when it mismatched the prime. This benefit could be enhanced even further when participants were not only primed perceptually, but also imitated the primes orally in a proto-song (e.g., saying “ba-ba-KA-ba-ba-KA” for a weak–weak–strong–weak–weak–strong sequence).

Ludke, Ferreira, and Overy (2014) found that participants engaged in a paired-associate phrase learning task in a foreign language (Hungarian) performed significantly better when they imitated melodic input in training (i.e., when they listened to sung utterances and sang them as well) than when they imitated plain prose or rhythmic speech. No parallel effect emerged when the task was performed in English, the participants’ native language. These findings suggest that sung input can enhance the verbatim recall of phonetic input when the input language is unfamiliar or the task is difficult (see Ludke et al. (2014) and Racette and Peretz (2007) for further discussion).

#### Effects of rhyme and melody on implicit language learning

Apart from short-term effects on verbal recall, a rhyming or melodic input presentation may have a positive effect on the acquisition of linguistic categories and sequences. After all, like prose, melodies and rhymes are organized temporally and incorporate variations in pitch, volume, and tone (Fonseca Mora, 2000; McMullen & Saffran, 2004), which serve as cues in language learning. Hence, it is conceivable that a melodic or rhyming presentation of linguistic input enhances language learning. Studies looking at musical and linguistic sequence learning in isolation demonstrate that adults and infants are sensitive to transitional probabilities in both domains. Such transitional probabilities are particularly informative regarding structural boundaries, and adults and infants appear to exploit them in order to segment speech and music alike (Aslin, Saffran, & Newport, 1998; Saffran, Aslin, & Newport, 1996; Saffran, Johnson, Aslin, & Newport, 1999; Saffran, Newport, & Aslin, 1996). For instance, in learning music and language, participants exploit prosodic cues, such as rhythm, stress, or intonation (Hirsh-Pasek et al., 1987; Jusczyk & Krumhansl, 1993; McMullen & Saffran, 2004). There is evidence to suggest that implicit learning of melodies creates auditory expectations (e.g., Tillmann & Poulin-Charronnat, 2010; for review see Ettlinger, Margulis, & Wong, 2011), and such expectations may be beneficial when the melodies accompany novel linguistic sequences, much like the rhythmic expectation effects on phoneme monitoring reviewed above (Cason & Schön, 2012; Ludke et al., 2014). Indeed, Schön et al. (2008) demonstrated that music can facilitate the acquisition of word boundaries. In their experiments, participants listened to a continuous stream of synthesized artificial three-syllable words that included no acoustic cues regarding the word boundaries. In order to segment individual words, participants had to rely on transitional probabilities, which were higher within words than across word boundaries. The stream was presented in either a synthesized spoken version (Experiment 1) or a synthesized sung version (Experiment 2), where each syllable was assigned a specific pitch. In the test phase, only those participants who had been exposed to a sung version of the stream were able to discriminate words from non-words. The positive effect of melodic input was still observed when the linguistic and melodic information was decoupled and variable syllable-pitch mappings were used, such that there was no correlation between linguistic and melodic transitional probabilities (Experiment 3). This suggests that a melodic presentation alone can facilitate language learning. Critically, however, the best results were attained by those participants who had received fully matched syllable-pitch mappings during training, suggesting that correlated transitional probabilities critically add to the positive effect of a melodic presentation.

With respect to rhyme, previous studies have indicated that rhyme awareness is associated with language abilities, such as articulation (Mann & Foy, 2007), phonological perception (Foy & Mann, 2001), and rhythmic awareness (Wood, 2006). There has also been growing support for a connection of rhyme awareness with reading and spelling abilities (e.g., Bryant, MacLean, & Bradley, 1990; Wood & Terrell, 1998). However, to date, no studies have evaluated the role of rhyming abilities for morphosyntactic learning.

To summarize, it is quite likely that meter, rhyme, and melody function as mnemonic aids (Wallace, 1994) and as aids in language processing and language learning: melodies provide cues to the length of lines in a song, the pitch contour within a line, and the number of utterance segments in a line. This can enhance verbatim recall and the detection of structurally relevant cues in the input. Rhymes provide most of these cues as well, being spoken rhythmically and with a proto-melodic pitch contour (Lehrdahl, 2001). Rhythmic speaking may be particularly beneficial for processing grammatically relevant words or parts of words, which, in prose, are often realized off-beat and in a phonetically reduced fashion. It is noteworthy that, in principle, both rhyme and melody allow for more variability in rhythm and pitch contour than prose. In children’s rhymes and songs, however, this variability is limited to the extent that specific rhythmic and melodic patterns are repeated across stanzas or lines.

Some authors have argued that presenting input in a metered, rhyming, or melodic fashion may provide *too much* information, overloading the linguistic signal (see Racette & Peretz, 2007; Wallace, 1994). However, if that were the case, rhymes and children’s songs would be much less popular than they are. Thiessen and Saffran (2009, p. 231) have suggested that “One possibility is that natural systems, such as music and language, present infants with a near-optimal level of complexity: enough to facilitate, but not enough to overwhelm. Natural musical and linguistic systems may have been shaped, by successive generations of learners, into a form that is well calibrated to infant learners.”

### The present study

The purpose of the present study was to examine how the combination of input structuring, rhyme, and melody affects the acquisition of gender-like subclasses in an artificial grammar that provides exclusively syntactic cues towards the gender-like subclass of any given noun. The artificial language contained three gender-like categories, as seen in German, and three case-like categories (see Table 2). It is noteworthy that most preceding studies have tested two instead of three gender-like categories, that is, the gender-case-like marker system we tested was more complex than most of the systems tested before. Furthermore, unlike in any of the preceding studies, there was considerable overlap in the markers featuring within and across the inflectional paradigms associated with each gender. Such syncretism is characteristic of the German gender-case system, which fills a total of twelve grammatical functions in the singular inflectional paradigms with only about half as many different forms (see Table 1 for the case of definite articles).

**Table 2:** The artificial gender-case-like markers used in Experiments 1A and 1B

Case-like category (locative markers)	Gender-like category		
	I	II	III
To-locative (nominative)	ino	ano	ono
By-locative (genitive)	gla	ino	gla
From-locative (dative)	sla	ino	sla

Following Brooks et al. (1993), the German case paradigm was replaced by a paradigm of locative markers (expressing to, by, and from). There were no noun cues as to the gender category membership of the nouns. In Experiment 1A, all sentences concerning one noun were presented in immediate succession as in Taraban (2004). This way, participants could mutually process all three case-like markers that belonged to one noun. In Experiment 1B, all sentences were presented in random order so that the sentences were no longer grouped together for each noun. This training method mimicked everyday life interactions, which are unlikely to feature the three case or case-like markers pertaining to one noun in immediate succession. In both experiments, participants were trained in one of the following four training modes: a) Prose, b) Rhyme, c) Melody, and d) Rhyme&Melody.

## Experiments 1A and 1B

### Method

#### Participants

Ninety-six participants (22 men), recruited at the Ruhr-University Bochum and University of Münster, Germany, took part in exchange for payment or course credits. All participants were German native speakers between the ages of 18 and 30 years (mean age 23.47 years, *SD* = 3.09 years, *N* = 92, with four participants not specifying their age in the post-experiment questionnaire). Participants were randomly assigned to one of eight groups, with each group consisting of 12 participants. There were no age differences between groups (*F* < 1) and female participants markedly outnumbered male participants in all groups.

#### Ethics, consent and permissions

Ethical approval was obtained from the Ethics committee of Ruhr-Universität Bochum as part of application number 4160-11. Upon signing up to the study, participants were informed about the general purpose of the experiment; their consent to participate was obtained in the first training session. At the end of the study, they were fully debriefed.

#### Materials

**Fig. 1:**
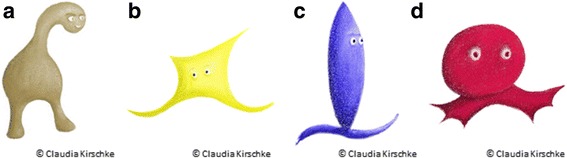
Examples of the fantasy creatures used in Experiments 1A and 1B. Tika (**a**) was the creature that acted as a subject in all stimulus sentences. Lelop, Filan, and Tekes (**b**–**d**) are three examples of a total of 36 different creatures featuring as objects in the stimulus sentences for the training and test sessions

For the language training, we created animated scenes of moving fantasy creatures in Microsoft PowerPoint. These animated scenes were combined with sentences describing the respective actions. In each scene, a single creature, “Tika” (see Fig. 1a), moved towards, around, or away from a variety of other fantasy creatures. Figure 1b, c, and d provide examples for three of these other creatures. All pictures depicting the fantasy creatures were created by Claudia Kirschke, who has kindly made the pictures available to us.

##### The lexicon

All elements of the lexicon were pseudowords in German with no orthographic German real-word neighbors. The pseudowords were generated using the program WinWordGen 1.0 for German (Duyck, Desmet, Verbeke, & Brysbaert, 2004). We took care that the pseudowords had a high bigram frequency. This ensured that that they were easy to pronounce for native speakers of German, i.e., they did not include overly complex consonantal combinations of more than two consonants. Despite their status as pseudowords, we shall refer to the individual lexical entries as nouns, verbs, and markers in order to indicate their grammatical function in the artificial language.

The lexicon of the artificial language contained 37 nouns referring to 37 corresponding fantasy creatures. There was one subject noun, “Tika”, which was the agent of all actions. The remaining 36 nouns depicted the patients of the actions, such as “Lelop”, “Filan”, or “Tekes” (see Fig. 1). They all featured in the grammatical function of an object, so these nouns will be referred to as *object nouns* in the following. Each object noun consisted of four to five letters (see Appendix 3 for an overview). They were pronounced as disyllabic words with four to six phonemes. We ensured that none of the object nouns included suffixes associated with a specific gender in German (e.g., -e, -el, -en, -er, -heit and -ung; see Wegener (1995) and Table 11 in Appendix 1). The object nouns and the creatures they depicted were assigned pseudorandomly to one of the three gender-like subclasses (I, II, III; see Table 2).^4^

Apart from the nouns, the lexicon included five grammatical markers carrying information about the gender- and case-like categories of the object nouns (see Table 2). As detailed previously, this set of markers differed from the sets of markers used in previous studies in that only five markers fulfilled a total of nine grammatical functions within the gender-case-like paradigm. Since German native speakers have already acquired a gender-case system in their first language, we made sure that the system of gender-case-like markers did not refer to thematic roles as do the German gender-case markers. Instead, our artificial markers expressed three locative conditions, with the choice of grammatical markers depending on the gender-like subclass of the object noun and the locative orientation of the subject noun’s (i.e., Tika’s) action. That is, a sentence was associated with a to-locative marker when Tika walked towards an object noun creature, it was associated with a by-locative marker when Tika walked around an object noun creature, and it was associated with a from-locative marker when Tika walked away from an object noun creature.

In addition to the nouns and the markers, the language included two verbs (*pim* and *pif*). *Pif* referred to the action of Tika moving towards or away from another creature, *pim* referred to Tika moving around another creature in a circle. As some gender-case-like markers featured more than once within the marker paradigm (see Table 2), these verbs ensured that all sentences differed from one another. For instance, the sentences describing the by- and from-locative movements for gender-like category II would be identical if only one verb had been used.

##### The language

**Fig. 2:**
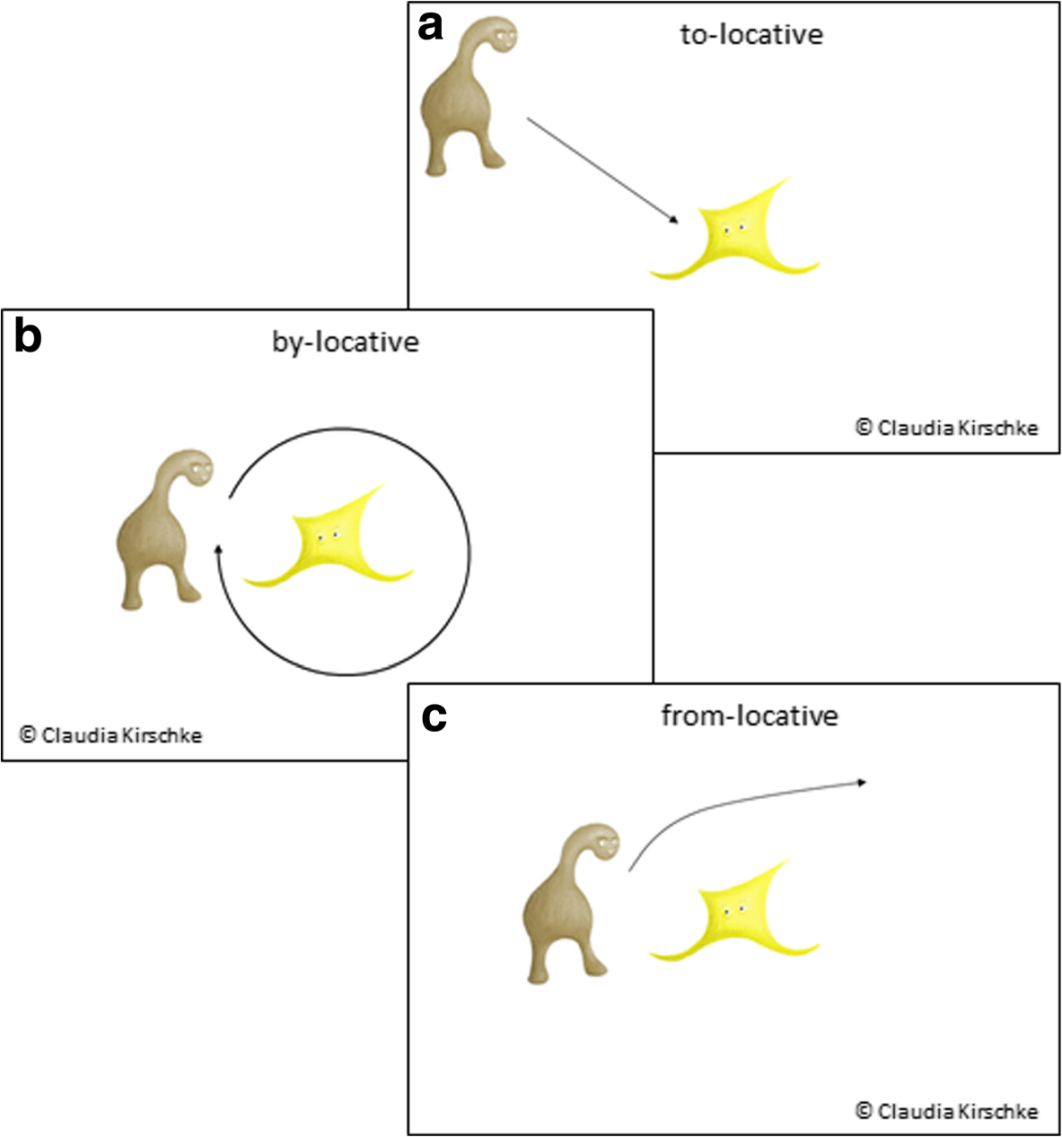
Examples of the animated scenes of Tika’s movements towards (**a**; to-locative), around (**b**; by-locative), or away from (**c**; from-locative) Lelop. For Lelop, the correct grammatical markers associated with each locative were ino, gla, and sla

We embedded the markers in sentences using the following syntactic structure: object noun–locative marker–subject noun (Tika)–verb. This sentence structure differed from the standard German sentence structure in the order of the syntactic units. Given the three case-like categories, each object noun was associated with three sentences expressing that Tika walks towards, around, or away from the object noun creature, respectively (see Fig. 2). For the creature “Lelop”, for example, which was assigned to the gender-like category I, the three sentences are shown in (4) to (6). In the linguistic glosses, we use G to refer to the respective gender-like category (I–III) and C to refer to the respective case-like category (I–III). 
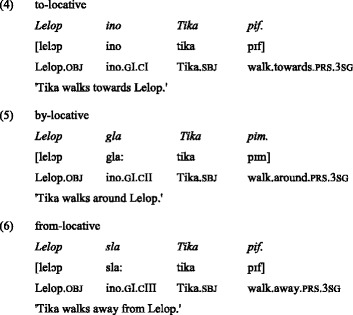

In the Rhyme condition, we used the same sentences and added a nonsense line rhyming with the last syllable of the sentence. As we had positioned the verbs at the end of the sentences and the verbs were the same for all object nouns, we were able to create identical nonsense lines for all object nouns, as exemplified below for “Lelop”: 
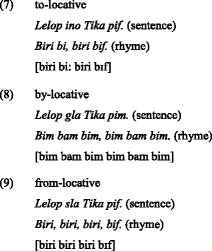


In conditions Melody and Rhyme&Melody, participants were exposed to a melodic versions of the non-rhyming and the rhyming training material, respectively, presenting all sentences (and rhymes, if applicable) in a sung version.

##### Creating and recording the training utterances

In order to prepare the stimuli to be used under the conditions Prose, Rhyme, Melody, and Rhyme&Melody, we asked a female professional singer and songwriter, Linda Kauffeldt, to compose the melodies for the sung conditions and to sing and record all utterances used in the training and test sessions of the experiment. In the Prose condition, she enunciated the utterances at a regular to slow speaking rate, articulating all vowels clearly without hyperarticulating them. As in a typical German declarative sentence, all pseudowords in the utterance were stressed on the first syllable, and pitch fell from the beginning to the end of the utterance. Figure 4 in Appendix 4 presents the average pitch contours for the to-, by-, and from-locative sentences in the Prose condition (red lines), based on a phonetic analysis for the items Lelop, Molun, and Gitun. These pitch contours were virtually identical across the to, by, and from sentences.

As we were not primarily interested in the effects of rhythm, the composer ensured that the three conditions Rhyme, Melody, and Rhyme&Melody shared the same rhythm. Each sentence had four beats (underlined in (10)), coinciding with the beginning of the creature’s name (e.g., “Lelop”), the marker (e.g., “ino”), “Tika”, and “pif”, respectively. Across the case-like variants of the training sentences, the creature’s name (e.g., “Lelop”) would always feature as an up-beat with the full beat (printed in fat letters in (10)) coinciding with the marker. 
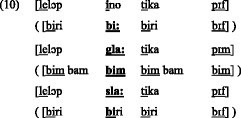


Figure 4 in Appendix 4 presents the pitch contour for the Rhyme condition (blue lines), based on a phonetic analysis for items Lelop, Molun, and Gitun. With Lelop (or any other creature’s name) being enunciated as an up-beat, pitch increased on the full beat, coinciding with the marker. Again, this pitch contour was virtually identical across the three case-like sentence types.

Figure 5 in Appendix 4 presents the melodies the composer created and sang for the conditions Melody and Rhyme&Melody, respectively. Again, the creature’s name functioned as an upbeat, with the full beat coinciding with the marker. In order to highlight the musical style of these conditions, the vocals were accompanied by a piano, which played the same tune as the singer.

In composing the melodies for the conditions Melody and Rhyme&Melody, the composer made sure that the structures of the sung sentences fit with standard structures of children’s songs, taking into account elemental common features of popular children’s music, such as repeatability, the use of major and minor harmonies, and a simple rhythm. These features support the transparency and accessibility of popular songs (Kramarz, 2007; see also Kauffeldt, 2011). Indeed, from a musical point of view, children’s songs lend themselves well to bringing together the components melody, meter, grammatical structure, and text content in an optimal way (Kauffeldt, 2011). The composer matched the two melodies for the groups Melody and Rhyme&Melody according to their form, structure, and musical quality (see Appendix 4). Note that based on the existing sentences, a six-lined structure was given for the Rhyme&Melody condition, while a three-lined structure occurred in the Melody condition (see examples for “Lelop” above). Since a six- or four-lined structure but no three-lined structure is common in children’s songs (see Appendix 2 for an example of a four-lined structure), we facilitated a four-lined structure for the Melody condition by embedding a nonsense line after the second sentence ([dubida: dubidu:]; see Figure 5a in Appendix 4).

When recording the utterances, we made sure that, within a given condition, the speed of delivery of the sentences was kept at a consistent rate by means of a metronome. For any given condition, the metronome speed was set to be at a typical speed of delivery for the training mode at hand, i.e., the speed was slightly faster in modes Prose and Rhyme than in modes Melody and Rhyme&Melody. Table 3 lists the duration of the recorded sentences. They were very similar across conditions, ranging from 2.15 to 2.98 s. However, due to the use of a metronome, the subtle differences in speed between conditions were very consistent. In an analysis of variance of the sentence durations measured in the four conditions, condition yielded a significant effect (*F*(3, 63) = 262.31; *p* < 0.001). There was no difference in sentence durations between the three gender-like subclasses and no interaction of condition and gender-like subclass (both *F*s < 1.15, *p* > 0.33).

**Table 3:** Duration of the recorded sentences (in seconds) by condition and gender-like subclass: mean, standard errors (SE), minimum (Min), and maximum (Max)

	Gender-like Category			All Items
	I	II	III	
Prose				
Mean	2.11	2.15	2.17	2.15
SE	0.017	0.022	0.042	0.017
Min	2.04	2.04	2.06	2.04
Max	2.18	2.25	2.42	2.42
Rhyme				
Mean	2.30	2.31	2.32	2.31
SE	0.013	0.016	0.018	0.009
Min	2.26	2.26	2.24	2.24
Max	2.35	2.40	2.42	2.42
Melody				
Mean	2.98	2.99	2.98	2.98
SE	0.010	0.010	0.005	0.005
Min	2.94	2.94	2.97	2.94
Max	3.02	3.02	3.00	3.02
Rhyme&Melody				
Mean	2.91	2.92	2.91	2.91
SE	0.011	0.016	0.014	0.008
Min	2.85	2.86	2.86	2.85
Max	2.95	2.98	2.99	2.99

To assess whether introducing a rhythmic mode of speaking induced any changes to the clarity and distinctiveness of the vowels enunciated by the speaker, we compared the vowel space of the vowels included in the six sentences for each of items Lelop, Gitun, and Molun in the Prose and the Rhyme condition, respectively (see Figure 6 in Appendix 4). There was virtually no difference in the vowel dispersion observed across the two conditions, suggesting that the rhythmic style of speaking in the Rhyme condition did not render the vowels more distinguishable. We were not able to perform parallel analyses for the conditions Melody and Rhyme&Melody, as the singer’s voice was accompanied by a piano in these conditions.

#### Design

Using a 2 × 4 design, we tested the effects of training method (blocked, random) and training mode (Prose, Rhyme, Melody, Rhyme&Melody). In Experiment 1A, we employed a blocked input presentation, that is, all sentences concerning one noun were presented consecutively. Thus, for example, participants were first exposed to all three sentences associated with the creature “Lelop” (see sentences (4) to (6) above), then to all three sentences associated with “Filan”, and so on, imitating the strophe-based structure of a children’s song, a poem, or a pun or language play. The sentences were presented in a fixed sequence, with Tika first walking towards, then around, and finally away from the object noun creature. In Experiment 1B, the training sentences were no longer grouped into sets of three sentences per creature. Instead, the sentences were presented in a random fashion. For example, first the to-locative sentence for “Filan” would be presented, next the from-locative sentence for “Lelop”, and so forth. Participants never saw the same object creature with Tika in a row and the locative relations were presented in a random order rather than in fixed sequences. Within each of the two levels of the factor training method, language training took place in one of the four different training modes. Training method and training mode were tested between participants.

#### Training and test procedure

In keeping with previous studies (Brooks et al., 1993; Taraban, 2004), the sentence training was preceded by a dedicated vocabulary training session (Session 1). After that, we administered two language training sessions using animated PowerPoint scenes and sentence recordings (Sessions 2 and 3) and one test session (Session 4). The choice of the tasks used in the training and test sessions was based on the relevant predecessor studies (e.g., Brooks et al., 1993; see Table 4 for an overview).

**Table 4:** Experiments 1A (blocked presentation in training) and 1B (random presentation in training): Overview of the tasks administered in the training sessions and the test session

	Exp. 1A	Exp. 1B
	Training (with feedback)	
Session 1	Vocabulary training	
Session 2	Vocabulary review	
	Blocked listen-and-repeat	Random-order listen-and-repeat
	Sentence action matching	
	Blocked sentence production	Random-order sentence production
Session 3	Blocked listen-and-repeat	Random-order listen-and-repeat
	Blocked sentence production	Random-order sentence production
	Random-order sentence production	
	Test (without feedback)	
Session 4	Vocabulary review	
	Random-order sentence production (old items)	
	Forced-choice grammaticality judgment (old items)	
	Random-order sentence production (novel items)	
	Forced-choice grammaticality judgment (novel items)	

Out of the 36 object nouns, 30 were used for training purposes. The sessions were conducted sequentially within 2 consecutive weeks, with two sessions of approximately 60 min each per week. Participants were instructed that they would learn an artificial language and that no further information about the language could be given until the debriefing after the completion of the study.

##### Session 1 (vocabulary training)

In Session 1, all 31 creature nouns were trained. At the beginning of the session, participants learned that they would get to know all fantasy creatures and were supposed to learn their associated nouns as a requirement for the next sessions. They were then exposed to the creature nouns in five blocks, each block containing six to seven different PowerPoint slides displaying one creature and its corresponding spoken and written name. Each block was trained separately. First, the items were introduced by twice presenting each picture of a single creature along with its spoken and written name. Subsequently, only images of the creatures were shown and participants were asked to name each of them from memory. Immediate auditory and orthographic feedback, i.e., the correct name, followed each response. This task was continued until participants were able to name all creatures in the block of creatures correctly. Then participants proceeded to the next vocabulary block. After completing all five vocabulary blocks, participants were given a set of cards, each showing one of the 31 creatures at the front and their written names at the back. Participants were instructed to review them at home and to return the cards to the experimenter at the beginning of the next session, which took place 2 or 3 days later. Apart from the written creature names used in Session 1, no further orthographic information was provided in the present study. All following syntactic items were provided aurally to ensure that our training method paralleled the conditions of first language acquisition as closely as possible.^5^

##### Sessions 2 and 3 (language training)

Session 2 started by testing the participants’ knowledge of the 31 creature nouns acquired during the first session. All participants reached the learning criterion of 95%, i.e., 29 correct responses. During all the following tasks administered in Sessions 2 and 3, 6 of the 30 object nouns were withheld in order to be used as novel-old items in the test session (see Appendix 3). As each object noun was associated with three sentences (see Materials), the tasks administered in Sessions 2 and 3 featured a maximum of 72 sentences in total. During all tasks, participants were instructed that they should provide the best answer they could, even if they were not sure of their response.

As the first task in Session 2, we administered a *listen-and-repeat task* (see Table 4). Participants listened to one or more sentences while watching the related actions, then repeated the sentence(s) and finally listened to the correct sentences as auditory feedback to monitor their response. In Experiment 1A, all sentences were presented in a blocked fashion, so participants listened to the three sentences associated with one object noun, and were then asked to repeat them and received auditory feedback for all three sentences. In Experiment 1B, featuring a random sentence presentation, participants were no longer required to memorize and repeat three sentences in a row, rather they repeated each sentence immediately and received immediate auditory feedback. Compared to a training mode that is fully analogous to the blocked condition and presents trios of randomly assorted sentences in the listen-and-repeat task, the presentation in a sentence-by-sentence manner should ease the processing load associated with memorizing the individual sentences. We surmised that, if anything, this should enhance participants’ training outcome, but that this enhancement would be unlikely to outweigh the advantage of the blocked over the random method of presentation.

In the second task of session 2 (*sentence-action matching*; see Table 4), the participants listened to 24 single sentences, one sentence per each object noun. Each sentence was presented along with a sample of pictures showing the three associated actions of the object noun creature. Participants were asked to select the corresponding image. The participants received immediate feedback on the correctness of the response via a red box marking the correct picture. The target sentence played with any given object creature was selected pseudorandomly, with each participant seeing 8 creatures each with a to, by, and from locative. The sentence-action matching task was administered identically in Experiments 1A and 1B, as there was only one test item per object noun and blocking of sentences by object creature was not possible.

Next, productive language training was introduced (see Table 4). Participants were instructed to watch an action and to generate the corresponding sentence for it. In Experiment 1A, all actions were presented grouped by object nouns (*blocked sentence production*). However, unlike in the blocked listen-and-repeat task administered earlier, participants produced the sentence for each action immediately and did not wait for the three actions for a creature to be completed. After each group of three sentences, the correct sentences were given as auditory feedback. In Experiment 1B, the actions to be described were presented in a pseudorandom fashion and participants were asked to produce the correct sentence to each action (*random-order sentence production)*. Feedback was given immediately after each sentence (see Table 4).

In Session 3, participants first completed the *blocked* or *random listen-and-repeat* and the *blocked* or *random sentence production tasks* once again, as described for Session 2 (see Table 4). After that, they all worked through a *random-order sentence production* task, that is, the task administered to the participants of Experiment 1B in Session 2 and early in Session 3 was now administered to all participants. By including it as the very last task in the last training session, we were able to compare participants’ performance on this task immediately after training and in the test session, which took place 2 or 3 days after the last training session (see Table 4).

##### Session 4 (test)

In the final session, which was identical for Experiments 1A and 1B, several tasks were administered to test participants’ mastery of the artificial language (see Table 4). It began with a vocabulary review of the 30 object creature nouns followed by four tests. In the first two tests, all stimuli pertained to the 24 object nouns the participants had worked with during training (old items).

First, we carried out a *random-order sentence production test*. It was almost identical to the last task administered in training, except that participants received no feedback and were exposed to only half of the trained material. The material comprised of the three actions pertaining to 12 of the 24 trained object nouns (four nouns per gender-like subclass), resulting in 36 actions in total. Next, participants performed a *forced-choice grammaticality judgment test*. They watched 36 actions associated with the remaining 12 trained object nouns that had not been used in the random-order sentence production test. All actions were combined with an aurally presented sentence. The action-sentence pairs were presented in random order. We instructed the participants to categorize the sentences regarding their grammatical correctness by assessing whether the sentence matched the action. The sentences were either correct (one third of all stimuli) or contained an incorrect marker in terms of a) the gender-like category of the noun (one third of all stimuli) or b) the case-like category (one third of all stimuli). To perform successfully in Tests 1 and 2, knowledge of the gender-like class of each object noun as well as the gender-case-like paradigms was necessary.

In the remaining two tests, participants were tested with novel, untrained sentences in order to test their ability to generalize the acquired gender-case-like paradigm to new object nouns. To this end, we employed the six object nouns not included in the training tasks, but reviewed regularly in the vocabulary review (novel-old object nouns). Additionally, six novel-new object nouns were tested which the participants had never seen or heard before (see Appendix 3). Two novel-new and two novel-old object nouns were assigned to each gender-like category. In the *novel + hint sentence production test*, participants were required to produce sentences to actions performed with the novel object nouns. Since the participants had no way of knowing the gender-like subclass of the novel nouns, we gave them a hint by aurally presenting the to-locative sentence of an object noun along with the corresponding action. Next, we asked the participants to generate the two remaining sentences (by-locative and from-locative) for the object noun along with the visual presentation of the actions. Finally, in the *novel forced-choice grammaticality judgment test*, participants listened to sentences pertaining to novel vocabulary. The sentences were presented in groups of three that each pertained to the same novel object noun. For each of the 12 novel object nouns, one correct and one incorrect group of sentences was presented, resulting in 24 test items in total. Incorrect test items included one incorrect sentence, which was either false concerning a) the gender-like category, b) the case-like category, or c) both, with each type of error featuring in one-third of the incorrect sentences. We asked participants to decide whether the group of sentences associated with one noun sounded grammatically correct or incorrect.

After the tests, we asked participants to fill in a questionnaire in order to find out about the level of awareness regarding the gender-case-like paradigm. Participants were asked questions on the structure of the acquired material to assess which aspects of the grammatical system they had learned and were able to report.

### Data analysis

In the production tests (*random-order sentence production* in Session 3 and the test session and *novel + hint sentence production* in the test session), errors were restricted to participants using the wrong markers. Other mistakes, namely the wrong use of an object or verb, occurred rarely (15 and 265 out of 6336 cases in Experiments 1A and 1B, respectively, corresponding to 0.24% and 4.18% of the data). They were not excluded from the analyses. There were no invalid trials; that is, all participants responded to all stimuli.

In a first set of analyses, we compared the participants’ accuracy with the chance level (50%) using binomial tests (*p* < 0.05 for all tests). We set this chance level following the analyses reported in previous studies (Frigo & McDonald, 1998; Kempe & Brooks, 2008; Taraban, 2004). It reflects that, with any answer a participant produces, he or she can produce the correct or an incorrect response, be that a sentence including a particular locative marker or a forced-choice grammaticality judgment.

Next, we assessed the effects of training method and training mode on participants’ performance. Marker accuracy was analyzed using linear mixed effects models as implemented in the lmer-function of the lme4 package (Bates, Maechler, Bolker, & Walker, 2015) for R (R Development Core Team, 2012), using a binomial link function and the Bobyqa optimizer. For all analyses, the full model supported by the design was used, including maximal random effects (Barr, Levy, Scheepers, & Tily, 2013), i.e., random intercepts for participants and items, as well as random slopes for items for the effects of training method, training mode, and their interaction. Training mode was contrast coded using sum coding with Prose functioning as the baseline and Rhyme, Melody, and Rhyme&Melody being compared to it. Following Barr et al. (2013), we used a model comparison approach. Based on the full model, we computed models excluding Blocking, Rhyme, Melody, Rhyme&Melody or the interactions of Blocking with each of the presentation modes, carrying forth the random slopes and intercepts of the full model. As the models included a large number of factors, they often failed to converge. For most of the models, convergence was reached when the random correlations were removed (see Barr et al., 2013). Occasionally, random slopes also had to be removed. In these cases, we removed the slope(s) that were associated with the least amount of variance.

### Results and discussion

In the groups of participants who had been trained with the blocked training method, all participants significantly exceeded chance (50%) in the random-order sentence production task carried out at the end of the last training session and in all tasks administered in the test session (binomial tests, *p* < 0.05), indicating that in Experiment 1A, all groups had learned the gender-case-like system of locative markers. Experiment 1B yielded a different pattern of results. In the random-order sentence production task participants completed at the end of the last training session, the marker accuracy of all four groups was significantly greater than chance. However, in the random-order sentence production tests that were administered in the test session with old and novel items, respectively, participants in the Prose group did not exceed the chance level. This result is compatible with the finding reported by Taraban (2004) that participants trained with a random presentation of the material in a prose mode did not exceed the chance level of 50%. Participants trained with the other three training modes exceeded the chance level. In the *forced-choice grammaticality judgment* tests, participants trained with the random training method performed significantly better than chance.

Next, we analyzed the effects of training method (blocked, random) and training mode (Prose, as compared to Rhyme, Melody, and Rhyme&Melody) for each task.

#### Old items

##### Production tasks

The results of the random-order sentence production tests with feedback (Session 3) and without (Session 4) are summarized in Table 5. Participants who had received blocked input during training significantly outperformed those receiving randomly structured input (see Table 6 for a summary of the results of the statistical analyses). Tables 5 and 6 also show that, compared to a Prose training mode, the modes Rhyme, Melody, and Rhyme&Melody significantly enhanced participants’ performance in both production tests. At both testing times, there was a significant interaction of training method and Rhyme&Melody, reflecting that, compared to Prose, the effect of Rhyme&Melody was particularly strong in the blocked as compared to the random training method. This increase was as high as 20% and 29% in Sessions 3 and 4, respectively, for the blocked training method, whereas it was much lower (11% in Session 3 and Session 4) with the random training method.

**Table 5:** Experiments 1A (blocked presentation in training) and 1B (random presentation in training): Percentages of correct markers (subject means with standard deviations) in the random-order sentence production task with old object nouns at the end of the last training session (with feedback) (SPwF), the random-order sentence production test with old object nouns in the test session (without feedback) (SP), the forced-choice grammaticality judgment task with old object nouns (GJ), the novel + hint sentence production task (SP, collapsed over novel-old and novel-new nouns), and the novel forced-choice grammaticality judgment task (GJ, collapsed over novel-old and novel-new nouns)

	Blocked presentation (Experiment 1A)					Random presentation (Experiment 1B)				
	Old items			Novel items		Old items			Novel items	
Training mode	Production		Comprehension	Production	Comprehension	Production		Comprehension	Production	Comprehension
	SPwF	SP	GJ	SP	GJ	SPwF	SP	GJ	SP	GJ
Prose	71.99 (16.73)	60.88 (16.43)	77.31 (8.36)	92.36 (19.29)	96.18 (6.52)	54.75 (17.94)	49.54 (19.56)	66.67 (9.77)	50.69 (17.48)	69.79 (10.97)
Rhyme	80.44 (16.46)	75.23 (20.01)	82.18 (11.87)	92.36 (10.93)	97.22 (2.71)	62.73 (15.27)	55.32 (20.67)	69.91 (9.31)	61.46 (19.39)	76.39 (8.94)
Melody	83.22 (13.14)	82.64 (14.48)	87.27 (12.50)	99.31 (2.41)	99.31 (1.62)	60.07 (16.92)	58.33 (16.15)	74.07 (9.79)	64.24 (19.98)	72.92 (15.64)
Rhyme&Melody	92.48 (10.62)	90.28 (19.43)	96.06 (5.09)	100 (0)	99.65 (1.2)	65.28 (19.14)	60.65 (18.83)	75.23 (10.95)	64.58 (26.62)	82.64 (13.74)

**Table 6:** Experiments 1A and 1B: Inferential statistics testing for the effect of a blocked as compared to a random training method and for the difference between the prose training mode and the three training modes Rhyme, Melody, and Rhyme&Melody in the sentence production (SP) and forced-choice grammaticality judgment tasks (GJ). SPwF refers to the sentence production task administered at the end of the last training session

Training condition	Old items						Novel items			
	Production				Comprehension		Production		Comprehension	
	SPwF^a^		SP^b^		GJ^c^		SP^d^		GJ^e^	
	*χ* ^*2*^(1)	*p*	*χ* ^*2*^(1)	*p*	*χ* ^*2*^(1)	*p*	*χ* ^*2*^(1)	*P*	*χ* ^*2*^(1)	*p*
Blocking	38.76	0.001 ***	28.95	0.001 ***	23.28	0.001 ***	60.10	0.001 ***	36.07	0.001 ***
Rhyme	4.43	0.035 *	5.35	0.021 *	1.97	0.161	0.07	0.789	1.14	0.286
Melody	3.78	0.052 ^(^*^)^	9.21	0.002 ***	9.27	0.002 **	6.96	0.008 **	5.68	0.017 *
Rhyme&Melody	17.62	0.001 ***	20.65	0.001 ***	26.86	0.001 ***	12.64	0.001 ***	13.41	0.001 ***
Blocking:Rhyme	0.01	0.916	0.47	0.494	0.15	0.701	1.85	0.174	0.02	0.888
Blocking:Melody	0.69	0.407	2.76	0.097	0.77	0.381	1.17	0.280	3.23	0.072 ^(^*^)^
Blocking:Rhyme&Melody	4.92	0.027 **	8.52	0.004 **	9.92	0.002 ***	4.56	0.033 *	2.19	0.139

^(^*^)^ *p* < 0.08, *p < 0.05, **p < 0.01, ***p < 0.001

^a^Full model’s AIC: 6543.5, BIC: 6639.3, excluding the random effects of Melody, Blocking:Rhyme, and Blocking:Melody

^b^Full model’s AIC: 3534.0, BIC: 3638.5

^c^Full model’s AIC: 2583.0, BIC: 2669.1, excluding the random effects of Melody, Blocking:Melody, and Blocking:Rhyme&Melody

^d^Full model’s AIC: 1674.8, BIC: 1766.6, excluding the random effects of Rhyme&Melody

^e^Full model’s AIC: 1312.2, BIC: 1409.8, excluding the random effects of Melody, Rhyme&Melody, Blocking:Rhyme, Blocking:Melody, and Blocking:Rhyme&Melody

##### Comprehension test

The analyses of the participants’ performance in the forced-choice grammaticality judgment test with old items yielded results largely parallel to those found for the production tasks (see Table 5). As shown in Table 6, there was a main effect of training method, with the blocked groups performing significantly better than the random groups. Compared to the Prose training mode, modes Melody and Rhyme&Melody, but not Rhyme, significantly improved participants’ performance (see Table 6). For Rhyme&Melody, this effect was enhanced further by a blocked training method.

To summarize, the analysis of the results for the old items showed that a blocked input presentation yielded significantly better learning outcomes than a random input presentation. The best results were attained when both rhyme and melody were added to the blocked training method.

##### Learning trajectories for old items

In supplementary analyses, we analyzed the types of errors participants made with the old items when producing sentences during the course of training and test, i.e., in the blocked/random-order sentence production task in Sessions 2 and 3, and the random-order sentence production tasks at the end of the last training session and in the test session (see Table 4). Four different error types were analyzed: a) an error in which the case-like category of the marker was correct, i.e., a marker from the correct locative category was produced, but the marker was from the wrong gender-like category (gender error); b) an error in which the marker was from the correct gender-like category but the case-like category was incorrect (case error); c) an error in which both the gender- and the case-like categories were incorrect (gender-case error); and d) an error that could be either a gender error, a case error, or a gender-case error (not classifiable). Table 7 summarizes the total number of each error type for each task, collapsed across experiments and training modes. Clearly, across sessions, participants mostly made gender errors, whereas other errors occurred at a lesser rate. Gender errors were dominant during all sessions and did not vanish towards the later sessions, indicating that, as we had anticipated, choosing the correct gender-like category in the production tasks constituted the most prominent problem for the learners. Therefore, all following analyses focus on gender errors.

**Table 7:** Number of error types produced in Session 2 (blocked/random-order sentence production), the first production task in Session 3 (blocked/random-order sentence production), the second production task in Session 3, and in the test session (random-order sentence production)

Session	Total number of items	Error type			
		Gender error	Case error	Gender-Case error	Not classifiable
Session 2	6912	2127 (31%)	133 (2%)	96 (1%)	1215 (18%)
Session 3 (1st)	6912	1365 (20%)	113 (2%)	82 (1%)	851 (12%)
Session 3 (2nd)	6912	1089 (16%)	93 (1%)	73 (1%)	666 (10%)
Session 4	3456	683 (20%)	45 (1%)	35 (1%)	358 (10%)

**Fig. 3:**
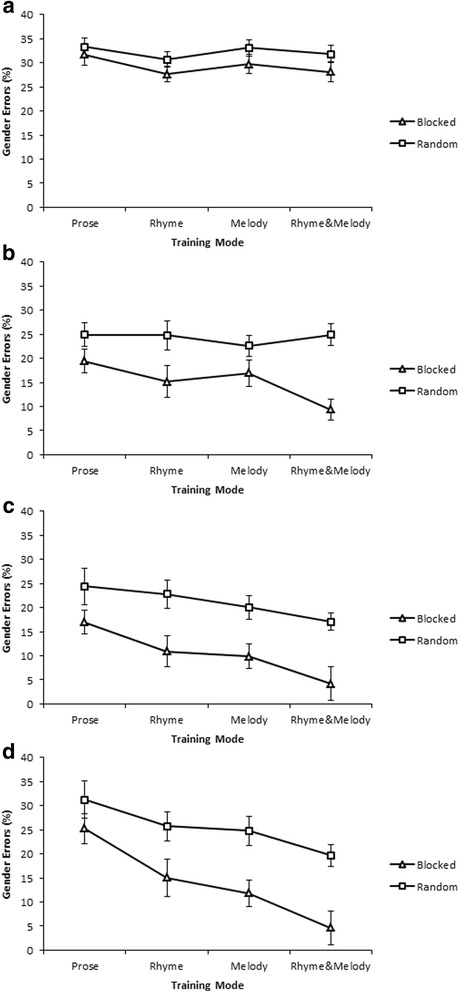
Mean percentages of gender errors of all responses produced in the blocked/random-order sentence production task (with feedback) in Sessions 2 (**a**) and 3 (**b**), the random-order sentence production (with feedback) at the end of the last training session (**c**), and the random-order sentence production in the test session (**d**)

**Fig. 4:**
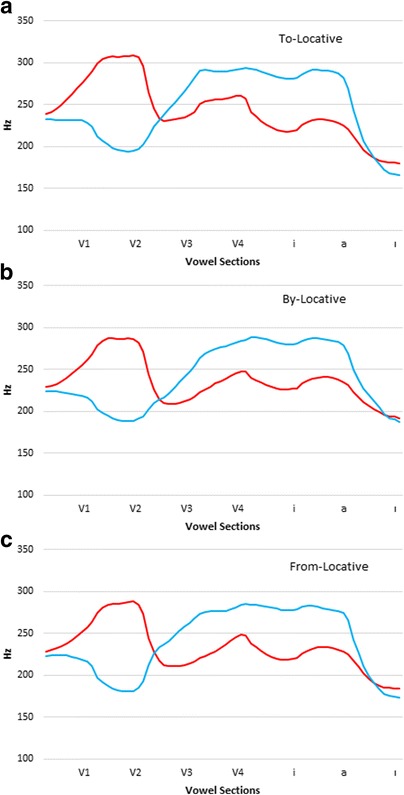
Pitch contours of the auditory stimuli used in training modes Prose (*red*) and Rhyme (*blue*). V1 and V2 are the two vowels in the object noun (Lelop, Molun, Gitun, V1: [e:, o:, i:], V2: [ɔ, u, u:]), and V3 and V4 are the vowels in the marker (ano, gla, ino, ono, sla; when the marker included only one long vowel ([a:] in gla, sla) instead of two shorter vowels ([a, i, o] in ino, ano, ono), the long vowel was mapped onto V3 and V4). [i], [a] and [ɪ] are the three vowels included in Tika and the verbs (pif or pim)

**Fig. 5:**
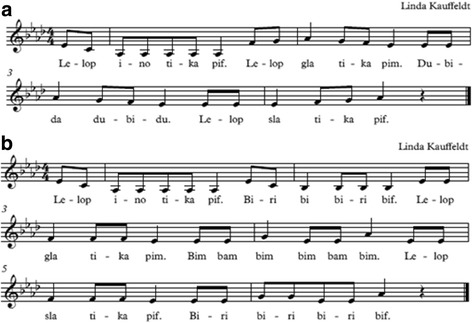
Melodies of the tunes used in training modes Melody (**a**) and Rhyme&Melody (**b**)

**Fig. 6:**
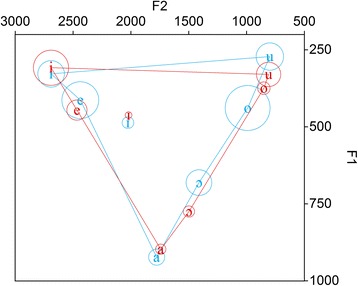
Vowel dispersion in the utterances used in training modes Prose (*red*) and Rhyme (*blue*)

Figure 3 depicts the percentage of gender errors out of all responses for all eight groups in each task. In order to assess the effects of session, training method, and training mode on the number of gender errors, we coded the errors as a binary variable (with all gender errors scoring 1 and all correct responses and all other errors scoring 0) and assessed the effects of training method and training mode for each session (see Fig. 3 and Table 8). As of session 3, there was an effect of training method, with participants trained with the random training method producing significantly more gender errors than those trained with the blocked training method.

**Table 8:** Results of the statistical analyses of gender errors with old items observed in Session 2 (blocked/random-order sentence production), the first production task in Session 3 (blocked/random-order sentence production), the second production task in Session 3, and in the test session (random-order sentence production)

	Session 2^a^		Session 3 (1st)^b^		Session 3 (2nd)^c^		Session 4^d^	
	*χ* ^*2*^(1)	*p*	*χ* ^*2*^(1)	*p*	*χ* ^*2*^(1)	*p*	*χ* ^*2*^(1)	*p*
Blocking	1.75	0.186	20.99	0.001 ***	31.16	0.001 ***	21.70	0.001 ***
Rhyme	3.39	0.066 ^(^*^)^	1.05	0.306	1.94	0.164	5.27	0.022 *
Melody	0.37	0.544	0.76	0.383	3.57	0.059 ^(^*^)^	8.11	0.004 **
Rhyme&Melody	1.92	0.166	5.34	0.021 *	16.68	0.001 ***	27.02	0.001 ***
Blocking: Rhyme	0.13	0.722	0.84	0.359	0.72	0.395	0.42	0.516
Blocking:Melody	0.23	0.634	0.01	0.960	0.51	0.476	1.64	0.201
Blocking:Rhyme&Melody	0.31	0.580	5.33	0.021 *	3.82	0.051 ^(^*^)^	6.16	0.013 *

^(^*^)^ *p* < 0.08, *p < 0.05, **p < 0.01, ***p < 0.001

^a^ Full model’s AIC: 5090.3, BIC: 5206.6

^b^ Full model’s AIC: 4668.0, BIC: 4784.3

^c^ Full model’s AIC: 4112.3, BIC: 4228.6

^d^ Full model’s AIC: 2285.3, BIC: 2389.8

With respect to the effects of training mode, we observed a significant advantage of mode Rhyme&Melody as of Session 3. From the start, this effect interacted with the training method, with the number of gender errors decreasing most clearly in the blocked Rhyme&Melody group (see Fig. 3). At the end of the study (Session 4), participants of the groups Rhyme, Melody, and Rhyme&Melody outperformed those of the group Prose (see Fig. 3).^6^

#### Novel items

As detailed in the Methods section, the production and comprehension tests were also carried out with six novel-old object nouns, which referred to creatures the participants had worked with during the vocabulary training but in no other part of the training phase, and with six novel-new object nouns, which participants had never encountered before.

Table 5 presents the test results of the novel + hint sentence production task and the novel forced-choice grammaticality judgment task, collapsed across novel-old and novel-new object nouns.^7^ As Tables 5 and 6 show, a blocked training method significantly increased participants’ levels of performance; in fact, the participants in the blocked condition performed near ceiling, yielding over 90% correct responses in the Prose condition. Nevertheless, the modes Melody and Rhyme&Melody increased participants’ accuracy rates significantly in both training methods. However, this increase was substantially smaller for the participants in the blocked training groups than those in the random-order training groups, simply because participants trained with the blocked method performed exceptionally well from the start. In the novel + hint sentence production task, this discrepancy yielded a significant interaction of training method and Rhyme&Melody training mode.

At first sight, it may seem odd that participants performed better overall in the tests with novel items than in the tests with old items (see Table 5), especially so in the sentence production task. However, since for the novel items participants had no way of telling their gender, participants were given a to-locative action for each novel creature and listened to the corresponding to-locative sentence. Next, they were asked to produce the by-locative and the from-locative sentences for this creature while watching the corresponding actions, so all they had to do was to retrieve the correct case-markers within a given gender-like marker paradigm. By contrast, in all tasks with old items, participants received no hints regarding the gender-like subclass of the individual object nouns. Thus, to perform well on the tests with old items, participants needed to not only use their knowledge about the paradigms of locative markers, but also assign the right gender-like category to each object noun. Therefore, provided the participants were aware of the marker paradigms associated with each gender-like category, the generalization tests with novel items were easier to complete than those with old items. The fact that participants of the blocked training groups performed exceptionally well from the start suggests that, by the time of the test session, they were fully aware of the composition of the three marker paradigms.

In summary, the results from Experiments 1A and 1B demonstrate that a blocked presentation is necessary and sufficient for the acquisition of a complex grammatical paradigm with three gender-like and three case-like subclasses, even when it includes substantial form overlap. In sentence production, participants in the group Rhyme&Melody consistently outperformed the participants in the Prose group, especially when being trained with structured input. This suggests that acquiring gender-like subclasses when no noun cues but only syntactic cues are available can be enhanced considerably by a combination of structured input, rhyme, and melody in training.

#### Questionnaire

To examine the extent of explicit knowledge participants were able to establish about the system of gender-case-like markers, we asked participants to fill in a questionnaire at the end of the study. They were asked to indicate whether they had noticed that the sentences followed certain rules and, if so, to write down those rules. The answers reflected four different types of paradigm knowledge: a) correct and full knowledge of the gender-case-like system, meaning that participants were able to describe which markers were used in connection with which action (e.g., “ino, ano and ono go along with walking towards a creature”) and which sets of markers belonged together (e.g., “ino goes along with gla and sla”) (cf. column ‘full’ in Table 9); b) complete knowledge of the case-like system only, i.e., participants were able to describe which markers occurred in connection with which actions, but could not describe which set of markers belonged to a shared gender-like category (column ‘locative’ in Table 9); c) incomplete knowledge of the gender- and case-like system, i.e., participants could describe an extract of the gender-case-like system (e.g., “ino goes along with walking towards a creature”) (column ‘partial’ in Table 9); and d) no knowledge of the paradigm at all. There were no participants with only partial knowledge of gender-like subclasses along with full mastery of the case-like system. Note that to classify as such, the participants would have had to indicate that they knew that one group of nouns was associated with markers ino, gla, and sla, another with markers ono, gla, and sla, and a third one with markers ano and ino. We believe that any participants managing this level of knowledge of the marker system would virtually always also know about the allocation of markers to case-like categories and hence master the full system (Type (a) of our classification).

**Table 9:** Questionnaire results: Type of gender-case-like paradigm knowledge for the four groups (n = 12) trained with the blocked training method (Experiment 1A) and the random training method (Experiment 1B), respectively

Training mode	Type of paradigm knowledge			
	Full	Locative	Partial	None
Blocked training method (Experiment 1A)				
Prose	8	3	1	0
Rhyme	8	3	1	0
Melody	11	1	0	0
Rhyme&Melody	11	1	0	0
Total	38	8	2	0
Random training method (Experiment 1B)				
Prose	0	3	7	2
Rhyme	0	6	6	0
Melody	2	6	4	0
Rhyme&Melody	3	5	3	1
Total	5	20	20	3

As Table 9 shows, most participants of the blocked training method provided a correct description of the full system. In contrast, most participants of the random training method showed explicit knowledge (in whole or partially) of the locative system only and failed to establish the whole paradigm. Three participants, all of whom had been trained with the random method, showed no evidence of any knowledge of the marker system. A chi-square test revealed a significant relationship of training method (blocked vs. random) and type of knowledge (full, locative, partial) (*χ* ^2^(2) = 45.15, *p* < 0.001), confirming the connection between training method and type of paradigm knowledge. The participants without any knowledge of the marker system were not included in this test because, in their group, the expected frequency was smaller than 5. As might be expected, the type of knowledge a participant was able to establish during training (full, locative, partial, none) had a significant influence on the number of correct markers produced in the random-order sentence production task with old items administered in the test session (see Table 10; *χ* ^2^(3) = 270.68, *p* < 0.001).

**Table 10:** Number of correct and incorrect markers produced in test 1 by paradigm knowledge (collapsed over Experiments 1A and 1B)

Markers	Type of paradigm knowledge			
	Full	Locative	Partial	None
Correct	1248	599	412	43
Incorrect	300	409	380	65

In conclusion, the answers given in the questionnaire indicate that most participants in the blocked training method gained complete knowledge of the marker paradigm, whereas most participants in the random training method acquired the locative system only or otherwise fragmented knowledge. Moreover, the results support the assumption that a more complete grasp of the system is associated with better levels of performance in the test.

## General discussion

The objective of this study was to examine whether different forms of input optimization can facilitate gender-like category induction when phonological cues are missing. We found that a blocked presentation led to significantly better results in comparison to a random presentation. This finding is consistent with Taraban’s (2004) findings. Furthermore, we found that the combination of a rhyming and melodic presentation facilitates gender-like category induction. This was most apparent in the blocked stimulus presentation, suggesting that the combination of highly structured input with rhyme and melody can be particularly useful in acquiring morphosyntactic paradigms and lexical-syntactic categories. While our starting point was the assignment of German nouns to gender subclasses, our findings are likely to apply to other languages with similarly opaque assignment systems of nouns to subclasses. In fact, they most likely also extend to other grammatical categories, such as verbs, provided that syntactic cues to grammatical subclasses are available and can be presented to language learners in an optimized fashion.

By demonstrating an effect of structured input presentation, we extend Taraban’s (2004, Experiment 3) original findings in several ways. First, using a more complex grammatical system, consisting of three rather than two gender-like categories, we show that structured input is effective. Second, our findings suggest that, with structured input, participants are able to deal with form overlap in the marker system, an aspect of grammatical category learning that has not been investigated in much detail yet for gender-like subclasses. Third, we were able to induce a substantial training effect using an oral, i.e., fleeting, presentation of the training sentences, as compared to the orthographic one in Taraban’s study.

Note, however, that our blocked condition differed slightly from that implemented by Taraban in that we presented the training sentences pertaining to one noun in the same order throughout (to-locative, by-locative, from-locative), whereas Taraban had varied their order. We had to use a fixed order due to the musical structure of the melodies used in the Melody and Rhyme&Melody modes. A specific melody was associated with each locative structure, resulting in a chorus-like melody when all three sentences were presented as a group. One might argue that our fixed order of presentation of the three locative sentences may have rendered learning somewhat easier for the participants in our study than for those in Taraban’s study. However, as we have detailed above, our artificial language and our training regime was, overall, more complex than Taraban’s due to the more complex gender-case-like paradigm and the auditory presentation of the training mterial.

Descriptively, Rhyme yielded the least consistent and Rhyme&Melody the most consistent increase in learning outcome over Prose, with Melody yielding an intermediate increase. Thus, even though the effect of Rhyme on its own was rather weak, it apparently helped to increase the effect of Melody in the Rhyme&Melody condition, especially in the blocked training method. We think that the advantage of Melody on its own over Rhyme on its own came about because Melody naturally affected all syllables of the training sentences. It is likely that this made the sentences, and especially the markers, more memorable in the melodic than in the rhyming condition. With the way we created the sentences and the rhyming line, Rhyme primarily highlighted the last syllable, i.e., the verb of the sentence. The verb distinguished sentences describing Tika walking towards or away from a creature (pif) from those describing Tika walking around a creature (pim), potentially highlighting this case-relevant distinction. However, this was clearly not sufficient to yield as strong training effects on gender-like category induction as those induced by Melody. When combined with Melody, however, the two forms of structuring clearly had synergetic effects, as participants trained with mode Rhyme&Melody were able to make use of both sources of highlighting the sentence structure and content and learnt fastest. It is also noteworthy that the analysis of gender errors showed that the emerging benefit of Rhyme&Melody over Prose was temporally correlated with the emerging benefit of blocking and the interaction of blocking and Rhyme&Melody, so clearly it was the combination of input optimization techniques that did the trick. Future research will have to show whether other forms of a rhyming presentation, for instance rhymes that highlight the relevant markers, yield different effects from the Rhyme condition implemented in our study, both in isolation and in combination with other forms of input optimization. Note, however, that such an implementation of Rhyme would mean that the rhyme lines differ across the gender-like subcategories, which we aimed to avoid in our study.

Before turning to the wider-reaching implications of our findings, we want to discuss two potential limitations of our study. First, the blocked and random training methods not only differed in terms of the order of presentation of the stimuli but also in the way participants engaged with them. In Experiment 1A (blocked presentation), participants listened to the three sentences associated with one object noun in a row, and were then asked to repeat them and received auditory feedback for all three sentences. By contrast, in Experiment 1B (random presentation), we did not mirror this mode of presentation by segmenting the random order of sentences into subgroups of three. Instead, participants repeated each sentence immediately and received immediate auditory feedback. One might argue that this reduced the processing load associated with memorizing the individual sentences, thus enhancing participants’ training outcome. Yet, despite this, we found that participants trained with the random training method were consistently less successful in acquiring the gender-like subclasses than the participants trained with the blocked training method.

Second, in the Rhyme and Rhyme&Melody modes used in Experiments 1A and 1B, every sentence was followed by a rhyme. Therefore, in these training modes, the presentation duration of each auditory stimulus was substantially longer than that of the stimuli in the modes Prose and Melody. One might argue that these differences played a role in acquiring the marker system. However, if that had been the case, we should see that the results of participants exposed to a rhyming version differ from those trained in a non-rhyming mode. Our results refute this prediction, as—descriptively—the differences in results between the effects of the modes Rhyme and Prose on participants’ learning outcome were no greater than those seen between the Prose and the Melody modes, which did not feature a rhyming line.

In summary, we have demonstrated that a structured input, presented in a rhyming and melodic fashion, is most effective in assisting the acquisition of gender-like subclasses. The training conditions we tested were designed to mimic everyday interactions with children in the form of language play or, more specifically, children’s songs on the one hand and less structured conversational interactions on the other. Our findings suggest that unstructured everyday interactions, presented in a prose fashion, might not be a sufficient basis for acquiring gender-like subclasses, whereas structured input, presented in a rhyming and sung fashion is such a basis. Hence, our findings make a strong case for using language play—nursery rhymes, children’s songs and related structured input, such as children’s books—for language learning (see also Good, Russo, & Sullivan, 2015). Input structuring can help learners focus their attention on relevant grammatical markers. Similarly, compared to a prose mode of delivery, where the markers are often realized off-beat and in a phonetically reduced fashion, a rhythmic or sung delivery of the input may be beneficial to processing grammatically relevant markers. In line with our findings, Kempe, Brooks, Mironova, and Fedorova (2003) have shown for Russian children that diminutivization, which is characteristic for child-directed speech in Russian (see Kempe, Brooks, Mironova, Pershukova, & Fedorova, 2007), is beneficial to gender acquisition, most likely because diminutives provide clearer and less confusable phonological cues towards a noun’s gender. Together, these findings suggest that child-directed speech and language play, especially children’s songs, may all enhance implicit morphosyntactic learning.

Note, however, that learning may not have been exclusively implicit. As apparent from the questionnaire results, most participants of our study were able to establish some kind of explicit knowledge of the marker paradigm. Participants in the blocked training method mainly showed knowledge of the complete marker system, whereas participants in the random training method mostly demonstrated partial knowledge. Thus, input structuring seems to support the establishment of complete paradigm knowledge. This result is consistent with the assumption that knowledge gained in artificial grammar learning can be partly consciously identified, even when explicit instructions are missing (e.g., De Jong, 2005; Pothos, 2007; Van den Bos & Poletiek, 2010).

There are several issues that need to be addressed in future studies. We have already pointed out that it would be worthwhile to assess different implementations of input optimization using rhyme. Another relevant parameter of input optimization that we did not manipulate systematically in our study is rhythm (see Selchenkova et al., 2014). Rhythm naturally accompanies rhyming and melodic structures. To further investigate the role of rhythm, a particularly rhythmical pronunciation of the prose mode input material could be used. Another issue worthy of evaluation would be the complexity of the grammatical system itself. Since we used a limited extract (3 gender-like categories × 3 case-like categories) of the German gender-case system, the next step would be to find out if similar results can be obtained when the system is made more complex, for instance by introducing number (singular, plural) to the paradigm or when the system is transferred to a different domain, such as arbitrary grammatical subclasses of verbs. Finally, it is necessary to conduct the present and all following studies not only with adults but also with children; after all, the aim is to identify the benefits of children’s poems and songs for language learning. Brooks et al. (1993) replicated the findings they reported with adults (Experiment 1) with 9- and 10-year-old children (Experiment 2). Ideally, though, a replication of the present study should involve even younger, pre-literate, children aged 4 or 5. For these children, singing and rhyming are the most natural forms of linguistic activity and, as we have pointed out in the Introduction, children should ideally have acquired the gender-case system of German (or any other gender-marking language) prior to learning to read and write. Note, however, that the phonological short-term memory of children at that age will be less developed than that of older children, so a challenge in testing such young children will be to simplify the language in such a way that they can process and memorize the sentences (Gathercole, Hitch, Service, & Martin, 1997).

### Implications

The present findings demonstrate that optimizing syntactic cues through input structuring can support the acquisition of artificial grammatical subclasses, especially when the input is presented in a rhyming and melodic fashion. In everyday language promotion situations, this combination naturally occurs in language play in general, and children’s songs in particular. There are some didactic concepts for pre-school and elementary school children in Germany that make systematic use of songs and other forms of language play in order to make complex morphosyntactic paradigms accessible (e.g., Frieg et al. 2012; Frieg, E. Belke, G. Belke, Hoffmann, Bebout, & Kauffeldt 2014; Kauffeldt et al., 2014). As with these concepts, teachers might compile their own language learning material aimed at combining the mechanisms tested in the present study (input structuring, rhyme, and melody) for facilitating grammar acquisition.

## Appendix 1

**Table 11:** Gender assignment regularities for german, as identified by Wegener (1995)

	Regularity	Examples	Exceptions
1	When unmarked, nouns ending in -e are feminine.	Hose (trousers.F), Jacke (jacket.F), Nase (nose.F)	Auge (eye.N), Ende (end.N), Löwe (lion.M) Käse (cheese.M)
2	When unmarked, monosyllabic nouns are masculine.	Kopf (head.M), Fuß (foot.M), Schrank (closet.M)	Hand (hand.F), Bein, (leg.N)
3	When unmarked, nouns ending in -el, -en, -er are masculine.	Löffel (spoon.M), Rücken (back.M), Finger (finger.M)	Messer (knife.N), Schulter (shoulder.F), Pendel (pendulum.N) Becken (basin.N, also pelvis.N)
4	The grammatical gender of labels for male and female creatures is masculine and feminine, respectively.	Mutter (mother.F), Vater (father.M)	Mädchen (see (2))
5	In the case of derivational nouns, the derivational suffix determines the noun’s gender.	Krank-heit (ill-ness.F), Heiz-ung (heat-ing. F/heat-er. F)	Ergeb-nis (result.N), Erkenn-t-nis (insight.F)

All examples and exceptions listed with regularities 1 to 3 and 5 are taken from Wegener (1995), except “Löffel”, “Messer”, and “Schrank”

## Appendix 3

**Table 12:** Artificial object nouns used in Experiments 1A and 1B

Item number	Object noun	Number of phonemes	Summated bigram frequency
1	elom	4	7787
2	teos	4	8214
3	riun	4	10,290
4	unak	4	9259
5	etef	4	10,409
6	ilil	4	7599
7	deluf	5	11,848
8	eitak	4	16,832
9	besak	5	11,047
10	refam	5	10,431
11	gunal	5	12,615
12	jetem	5	10,741
13	gitun	5	13,482
14	lelop	5	11,810
15	urets	5	12,582
16	osret	5	11,122
17	ketiz	6	10,727
18	atunz	6	12,051
19	filan	5	1438
20	molun	5	10,379
21	tekes	5	14,104
22	geop	4	6572
23	tetak	5	13,261
24	inut	4	7084
25	melam	5	12,564
26	witim	5	11,101
27	akest	5	13,904
28	rebif	5	10,397
29	unol	4	8837
30	noref	5	11,274
31	astop	5	10,505
32	imres	5	11,300
33	abun	4	8898
34	runot	5	10,805
35	elwan	5	11,828
36	nelun	5	16,103

Items 31 to 36 (novel-new object nouns) were not used during the training procedure. Items 25 to 30 (novel-old object nouns) were only used during the vocabulary training and vocabulary review, but not during the syntax training

## Appendix 4

### Acoustic properties of the training utterances

In order to characterize the phonetic properties of the stimuli used in the training modes Prose and Rhyme, we had a trained phonetician assess their pitch contour and the degree of vowel dispersion (see Figs. 4 and 6, respectively). This analysis was based on the three sentences per condition for each of the items Lelop, Molun, and Gitun. A parallel analysis of the corresponding stimuli in the sung conditions (Melody and Rhyme&Melody) was not possible, as these stimuli were accompanied by a piano, whose tune overlaid the speech signal. To provide information about the pitch contour of these stimuli, we have included the music sheets displaying the melodies sung in conditions Melody and Rhyme&Melody (Fig. 5).

## Footnotes

1 The only exception to this are probably diminutivizing morphemes, such as “NOUN-chen” and “NOUN-lein”, which mean ‘little NOUN’ and often feature in child-directed speech. They transform nouns of any gender into neuter nouns, as in *Stempel* (stamp.M) and *Stempel-chen* (stamp-DIM;N), “little stamp”.

2 The natural gender principle states that the grammatical gender of labels for male and female creatures is masculine and feminine, respectively.

3 We assessed the predictive value of the gender assignment rules put forward by Wegener (1995) by applying them to 222 object names taken from the Snodgrass and Vanderwart (1980) picture database. These object names had been rated previously for their age-of-acquisition (Schröder, Kauschke, & De Bleser, 2003). We observed that 71% of the object names could be unambiguously assigned to the correct gender based on Wegener’s (1995) rules. It is noteworthy, however, that this proportion dropped to 56% when we considered only those nouns that had an age of acquisition of 3 years or younger, which is the age at which most children with German as their native language master the gender system (Szagun et al. 2007).

4 In a norming study, we asked 21 native speakers of German (students of Ruhr-University Bochum, mean age 24.9 years) to inspect the creatures and to judge spontaneously whether they considered the creature as male, female, or neuter by selecting the definite article that they would spontaneously associate with a given creature. Subsequently, we presented the same participants with audio recordings of the German pronunciations of the creature nouns and asked them to note for each stimulus how they thought it would be written in German and which definite article (der, die, das) would most likely go with it. Those creatures and object nouns that scored higher than 50% with one specific gender (e.g., with the masculine gender) were assigned in equal shares to all three gender-like categories of the language. The remaining object nouns were allocated randomly to all three gender-like categories resulting in groups of 12 object nouns per gender-like category.

5 We used written names in Session 1 for several reasons. First, we expected that this would render vocabulary acquisition easier for participants, as the same input was provided via two linguistic modalities. After all, we were not interested in the participants’ performance in vocabulary acquisition as such, but in how well they acquired the assignment of creature names to gender-like subclasses. Second, introducing the written names allowed us to use them for the participants’ individual review of the creatures and their names at home, between Sessions 1 and 2. Finally, there is evidence that hearing “known” words in a stream of pseudowords facilitates statistical learning (Cunillera, Càmara, Laine, & Rodríguez-Fornells, 2010), and we expected that knowing the creature names not only phonetically but also orthographically would render it easier for participants to segment the training sentences. Again, we were not interested in how well they learnt to segment the speech stream but in how well they acquired the assignment of creature names to gender-like subclasses.

6 In order to assess the interaction of time, training mode, and training method, we carried out an overall analysis of the number of gender errors observed from Sessions 2 to 4. For reasons to do with the way the data were coded, we were unable to include items as a random variable. This may be the reason why the AIC and BIC of the full model were rather poor (AIC: 23,828.5; BIC: 23,982.3) compared to those obtained in analyses including the full or at least some of the random effects structure for items. The analyses yielded significant main effects of time and training method as well as their interaction (all *χ* ^*2*^(1) > 30, *p* < 0.001). The modes Melody and Rhyme&Melody were associated with considerably fewer gender errors than the Prose mode, and this effect intensified over sessions (Session:Melody, *χ* ^*2*^(1) = 5.29, *p* < 0.05, Session:Rhyme&Melody, *χ* ^*2*^(1) = 24.39, *p* < 0.001). As we had seen in the overall analysis of accuracy, the effect of Rhyme&Melody was particularly pronounced in the blocked training method as compared to the random method (*χ* ^*2*^(1) = 8.11, *p* < 0.01). Most importantly, the three-way interaction of time, blocked presentation, and Rhyme&Melody was significant, reflecting that the beneficial effect of a blocked presentation in the Rhyme&Melody mode built up over sessions (*χ* ^*2*^(1) = 8.43, *p* < 0.01; see also Fig. 2).

7 In supplementary analyses of each training method, we assessed whether familiarity with the nouns (novel-new vs. novel-old) impacted on the participants’ performance by including familiarity, training mode, and their interactions as fixed factors in a linear mixed model as well as their random slopes (by participants). Familiarity did not significantly affect the results in the novel + hint sentence production task (Experiment 1A: *χ* ^*2*^(6) = 5.85, *p* > 0.4; Experiment 1B: *χ* ^*2*^(6) = 0.79, *p* > 0.9) or the forced-choice grammaticality judgment task (Experiment 1A: *χ* ^*2*^(6) = 7.92, *p* > 0.2; Experiment 1B: *χ* ^*2*^(6) = 3.63, *p* > 0.7), which is why we did not include familiarity in the analyses of training method and training mode across experiments.
